# Genetic alterations of malignant pleural mesothelioma: association with tumor heterogeneity and overall survival

**DOI:** 10.1002/1878-0261.12651

**Published:** 2020-03-10

**Authors:** Lisa Quetel, Clément Meiller, Jean‐Baptiste Assié, Yuna Blum, Sandrine Imbeaud, François Montagne, Robin Tranchant, Julien de Wolf, Stefano Caruso, Marie‐Christine Copin, Véronique Hofman, Laure Gibault, Cécile Badoual, Ecaterina Pintilie, Paul Hofman, Isabelle Monnet, Arnaud Scherpereel, Françoise Le Pimpec‐Barthes, Jessica Zucman‐Rossi, Marie‐Claude Jaurand, Didier Jean

**Affiliations:** ^1^ Centre de Recherche des Cordeliers Inserm Sorbonne Université Université de Paris Functional Genomics of Solid Tumors laboratory France; ^2^ Programme Cartes d'Identité des Tumeurs (CIT) Ligue Nationale Contre Le Cancer Paris France; ^3^ Institut de Pathologie Centre de Biologie‐Pathologie CHRU de Lille France; ^4^ Université de Lille France; ^5^ Laboratoire de Pathologie Clinique et Expérimentale (LPCE) et Biobanque (BB‐0033‐00025) CHRU de Nice France; ^6^ FHU OncoAge Université Côte d'Azur Nice France; ^7^ Assistance Publique‐Hôpitaux de Paris Hôpital Européen Georges Pompidou Paris France; ^8^ Service d'Anatomopathologie et Cytologie Hôpital Européen Georges Pompidou Paris France; ^9^ Service de Chirurgie Thoracique Hôpital Calmette – CHRU de Lille France; ^10^ Service de Pneumologie et Pathologie Professionnelle Centre Hospitalier Intercommunal de Créteil France; ^11^ Service de Pneumologie et d'Oncologie Thoracique Hôpital Calmette – CHRU de Lille France; ^12^ Réseau National Expert pour le Mésothéliome Pleural Malin (MESOCLIN) Lille France; ^13^ Service de Chirurgie Thoracique Hôpital Européen Georges Pompidou Paris France; ^14^ Present address: Service de Chirurgie Générale et Thoracique CHU de Rouen Rouen F‐76000 France; ^15^ Present address: Laboratoire de Biochimie (LBC) CNRS UMR8231 Chimie Biologie Innovation ESPCI Paris PSL Research University Paris F‐75005 France; ^16^ Present address: Service de Chirurgie Thoracique et Transplantation Pulmonaire Hôpital Foch Suresnes F‐92150 France

**Keywords:** gene mutations, prognosis, thoracic cancer, tumor heterogeneity, tumor molecular classification

## Abstract

Development of precision medicine for malignant pleural mesothelioma (MPM) requires a deep knowledge of tumor heterogeneity. Histologic and molecular classifications and histo‐molecular gradients have been proposed to describe heterogeneity, but a deeper understanding of gene mutations in the context of MPM heterogeneity is required and the associations between mutations and clinical data need to be refined. We characterized genetic alterations on one of the largest MPM series (266 tumor samples), well annotated with histologic, molecular and clinical data of patients. Targeted next‐generation sequencing was performed focusing on the major MPM mutated genes and the *TERT* promoter. Molecular heterogeneity was characterized using predictors allowing classification of each tumor into the previously described molecular subtypes and the determination of the proportion of epithelioid‐like and sarcomatoid‐like components (E/S.scores). The mutation frequencies are consistent with literature data, but this study emphasized that *TERT* promoter, not considered by previous large sequencing studies, was the third locus most affected by mutations in MPM. Mutations in *TERT* promoter, *NF2,* and *LATS2* were more frequent in nonepithelioid MPM and positively associated with the S.score. *BAP1*, *NF2*, *TERT* promoter, *TP53,* and *SETD2* mutations were enriched in some molecular subtypes. *NF2* mutation rate was higher in asbestos unexposed patient. *TERT* promoter, *NF2,* and *TP53* mutations were associated with a poorer overall survival. Our findings lead to a better characterization of MPM heterogeneity by identifying new significant associations between mutational status and histologic and molecular heterogeneity. Strikingly, we highlight the strong association between new mutations and overall survival.

## Graphical abstract

Our comprehensive genetic characterization of key‐altered genes including *TERT* promoter in malignant pleural mesothelioma led to the identification of new significant associations between the mutational status and the histological and molecular classifications. Our findings allow a better understanding of the genetic landscape in the context of tumor heterogeneity and highlight the high prognostic value of gene mutations in mesothelioma.

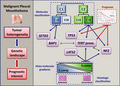

**Abbreviations**
MMB: biphasic MPM
MMD: desmoplastic MPM
MME: epithelioid MPM
MMS: sarcomatoid MPM
MPM: malignant pleural mesothelioma
NGS: next‐generation sequencing
TERT_prom: TERT promoter
TSG: tumor suppressor gene

## 1 Introduction

Malignant pleural mesothelioma (MPM) is a rare, severe, and rarely curable tumor arising in the pleura. MPM development is associated with occupational asbestos exposure that is the main etiological factor and remains a major public health concern even in countries that have banned asbestos. The evolution of our knowledge about tumor pathology taught us that MPM, such as other tumor types, presents specific molecular specificities for each patient. The lack of effective curative treatment for this cancer highlights the need to improve our knowledge of molecular alterations in the context of MPM heterogeneity with the aim to further design adapted therapeutic strategies and to implement precision medicine for this cancer.

The heterogeneity of MPM between patients was described at the clinical, histologic, and molecular levels. Histology defines three major types: epithelioid (MME), sarcomatoid (MMS), and biphasic (MMB). However, this classification in three types partially reflects the tumor heterogeneity at both the molecular and clinical levels (Jean *et al.*, 2012), and different histologic subtypes are also described (Husain *et al.*, 2013). Recent researches based on large‐scale genomic studies have identified molecular subtypes that go beyond the histologic classification (Bueno *et al.*, 2016; de Reynies *et al.*, 2014; Hmeljak *et al.*, 2018). A first classification in two transcriptomic subtypes (C1 and C2) allowed separating MME, the most frequent histologic type, according to patient outcome, the MME with a better prognosis being classified in C1 and the MME with a worse prognosis in C2 (de Reynies *et al.*, 2014). Classifications in four transcriptomic subtypes were also proposed and were associated to histology and prognosis (Blum *et al.*, 2019; Bueno *et al.*, 2016). The study based on The Cancer Genome Atlas (TCGA) data also identified four novel prognostic subgroups of MPM (iClusters 1–4) using an integrative multi‐omics classification (Hmeljak *et al.*, 2018). More recently, we described a new way to take into account MPM heterogeneity based on a deconvolution approach, which allows to define epithelioid‐like and sarcomatoid‐like entities and to determine their proportions, the E.score and the S.score, respectively, in a given tumor sample. These E/S.scores are highly associated with the prognosis and may have an impact on personalized therapeutic strategies in MPM, particularly targeted therapies and immunotherapies (Blum *et al.*, 2019). A recent publication has also shown that MPM heterogeneity is well described by a continuum (Alcala *et al.*, 2019).

A further step requires the integration of somatic mutations in the histologic and molecular profiles of tumors will permit to take into account genetic alterations of key genes, extend our biological information on the tumors, and improve the transfer to clinical practices (Horlings *et al.*, 2015; Huntsman and Ladanyi, 2018). The genetic landscape of MPM, recently specified by next‐generation sequencing studies (NGS) (Bueno *et al.*, 2016; Hmeljak *et al.*, 2018), confirmed the complexity and the heterogeneity of MPM mutation profiles between patients already suggested by previous sequencing studies (Andujar *et al.*, 2016). However, the links between the mutation profile and, on one hand, the clinical characteristic of patients and, on the other hand, MPM heterogeneity are not clearly established. Some associations between specific gene mutations and clinical data or histologic and molecular subtypes have been already described, such as the association of *TP53* mutations with survival (Bueno *et al.*, 2016), *TERT* promoter (*TERT*_prom) mutations with histology (Tallet *et al.*, 2014), or *BAP1* mutations with the C1 and the iCluster 1 subtypes, both enriched in MME tumor (de Reynies *et al.*, 2014; Hmeljak *et al.*, 2018). However, these associations deserved to be validated and new associations explored in independent large MPM series.

In order to refine the association between mutations and clinical data and to better define the MPM heterogeneity at the genetic level, we made a deep characterization of the mutations in 21 genes of interest selected based on literature data including the major MPM mutated genes (*BAP1*, *NF2*, *TP53*, *SETD2*, *LATS2,* etc.) and the *TERT_*prom using a collection of 266 MPM tumor samples with extended clinical annotations. To characterize the association between the mutations and the molecular heterogeneity described in MPM, tumor samples of the same cohort were classified into 2 and 4 subtypes, and the proportions of molecular components E/S.scores were determined.

## 2 Materials and methods

### 2.1 Frozen MPM tumors samples

The Inserm series was exclusively composed of frozen MPM tumor samples collected from 266 patients from biobanks of French hospitals (CHRU of Lille and Nice, Hôpital Européen Georges Pompidou of Paris and Centre Hospitalier Intercommunal of Créteil) linked to the French Mesobank network and certified by Mesopath as MPM (Galateau‐Salle *et al.*, 2014). This tumor collection (Inserm series) included biopsies or surgery resections of patients diagnosed between 2001 and 2017. The experiments were undertaken with the understanding and written consent of each subject. The study methodologies were conformed to the standards set by the Declaration of Helsinki and approved by a local medical ethics committee (CPP Ile‐de‐France II). The collected samples were registered in a database (DC‐2016‐2771) validated by the French Research Ministry. Samples were annotated with detailed clinicopathological and epidemiologic information obtained from pathology reports (Table 1; Table S1). Overall survival was calculated based on the initial date of diagnosis. The percentage of tumor cells in MPM samples was estimated by histologic examination by the pathology department of each hospital. For 21 patients, it was possible to obtain several tumor samples from diagnostic biopsies, surgery resections (Extended Pleurectomy/Decortication or Extrapleural pneumonectomy) and tumor samples of recurrence. MPM primary cell lines were established in our laboratory from 12 tumor samples.

**Table 1:** Clinico‐pathological and epidemiologic characteristics of the Inserm series of MPM patients. EP, extrapleural pneumonectomy; PD, pleurectomy with decortication; AR, atypical resection.

	Patients (*n* = 266)
Gender, *n* (%)	
Male	203 (76)
Female	63 (24)
Age (years)	
Median ± SD	69.0 ± 10.9
Range	20–91
Histology, *n* (%)	
Epithelioid	201 (78)
Biphasic	30 (12)
Sarcomatoid	21 (8)
Desmoplastic	5 (2)
Lymphohistiocytoid	2 (1)
Asbestos exposure, *n* (%)	
Exposed	186 (81)
Nonexposed	45 (19)
Tobacco consumption, *n* (%)	
Smoker	142 (55)
Nonsmoker	116 (45)
Stage IMIG, *n* (%)	
I	5 (2)
II	32 (14)
III	99 (45)
IV	86 (39)
Surgical treatment, *n* (%)	
EP	70 (26)
PD	36 (14)
AR	8 (3)
None	152 (57)
Chemotherapy treatment, *n* (%)	
Yes	189 (77)
No	56 (23)
Survival status, *n* (%)	
Deceased patients	204 (82)
Alive patients	46 (18)
Survival (months)	
Median	19.8
Range	0.1–178.3

### 2.2 MPM primary cell cultures

Malignant pleural mesothelioma primary cell lines were established in our laboratory from 12 tumor samples included in the Inserm series and cultured based on a previous established protocol (Zeng *et al.*, 1993). Briefly, fresh MPM tumor samples were reduced into pieces of less than 0.5 mm^3^ with a scalpel and transferred to a 24‐well tissue‐culture plate (TPP, Dutscher, Issy‐les‐Moulineaux, France) for adhesion in culture medium containing RPMI 1640, GlutaMAX™, HEPES buffer supplemented with 10% fetal bovine serum and 1% Penicillin‐Streptomycin (Gibco, Thermo Fisher Scientific, Villebon sur Yvette, France). Cultures were examined with a phase‐contrast microscope to detect monolayer growth. When the cells were confluent, a trypsin‐EDTA mixture (Trypsin/EDTA 0.05% in PBS; Gibco, Thermo Fisher Scientific) was used to detach the cells that were then amplified first in a 25‐cm^2^ and then in a 75‐cm^2^ flask (TPP). Cells were subcultured approximately every 2 weeks, depending on the cell line. MPM primary cell cultures were frozen in complete RPMI medium described before supplemented with 10% dimethyl sulfoxide (DMSO). When necessary, the cells are thawed rapidly at 37 °C, washed with 15 mL of complete RPMI medium, centrifuged, and cultured in flasks. The medium is changed after 24 h and then every 3 days. The cultures were used between passages 6 and 10.

### 2.3 DNA and RNA extraction

Genomic DNA and total RNA were extracted using a standard isopropanol precipitation procedure and Trizol (Thermo Fisher Scientific), respectively, or using the AllPrep DNA/RNA/miRNA Universal Kit (Qiagen, Courtaboeuf, France) according to the manufacturer's protocol.

### 2.4 Primer design for targeted sequencing

The design of the primers was performed on the genome assembly GRCh37 (hg19). The primer pairs specific for each gene were designed to amplify regions of up to 300 bp covering all the exon sequences at least twice with overlapping amplicons and thus at least 150 bp of adjacent intron sequences on either side using the primer3 program (Untergasser *et al.*, 2012). The primers were also tested by PCR *in silico* (UCSC) and SNPcheck3 (https://secure.ngrl.org.uk/SNPCheck/snpcheck.htm) to contain no SNPs with a frequency greater than 5% in the general population. Universal adapter sequences (one for sense primers and another for antisense primers) were added to each primer used for library preparation and sequencing.

### 2.5 Gene sequencing

Genomic DNA was quantitated using Hoechst dyes and a microplate reader. Gene sequencing was performed on a MiSeq^®^ System (Illumina, Evry, France). Targeted sequencing focused on 21 genes and the *TERT*_prom (Table S2) with an expected coverage of 400×. Only tumor samples with tumor content at least of 10% after histologic examination were used. Two types of libraries were generated by PCR, one covering the entire target regions (‘whole library’), the other specific to GC‐rich regions difficult to amplify (‘GC‐rich library’). The protocol of sequencing was established in our laboratory and slightly modified compared to the published protocol (Calderaro *et al.*, 2017). For each library (whole and GC‐rich), 300 ng of DNA was air‐dried at room temperature. Enrichment was performed on a Fluidigm Biomark^®^ instrument using ROX dye, Fluidigm loading reagent, and either TaqMan^®^ preamp master mix for whole library or Qiagen Multiplex PCR kit and DMSO for GC‐rich libraries. The primers pairs (1715 and 316 for the whole and the GC‐rich libraries, respectively) were multiplexed at 36 primer pairs per well for the whole library and seven for the GC‐rich library. Amplification programs were as follows: 95 °C 5′, 98 °C 2′, and during 16 cycles: 98 °C 15″, 60 °C 4′ for whole library, and 94 °C 15′ then three times two cycles: 94 °C 15″, 62 °C (61 °C and 60 °C for the next rounds) 30″, 72 °C 2′ then two times three cycles: 94 °C 15″, 59 °C (58 °C for the next round) 30″, 72 °C 2′ then two times four cycles: 94 °C 15″, 57 °C 30″ (56 °C for the next round), 72 °C 2′, then 72 °C 5′ for GC‐rich library. After purification with Agencourt AMPure^®^ XP magnetic beads (Beckman‐Coulter, Villepinte, France), products were PCR amplified with adaptors containing unique index sequences and sequencing adaptors (P5/P7 sequences) on a GeneAmp^®^ PCR 9700 system (Applied Biosystems, Thermo Fisher Scientific). Amplification programs were as follows: 98 °C 30″, during 7 cycles: 98 °C 10″, 60 °C 20″, 72 °C 60″ then 72 °C 5′ for whole library, and 94 °C 15′, during 12 cycles: 94 °C 15″, 60 °C 30″, 72 °C 2′ then 72 °C 5′ for GC‐rich library. A second sizing procedure was performed, and PCR products were quantified (relative quantification) on an Applied Biosystems^®^ 7900HT Fast Real‐Time PCR System with Syber Green^®^ and probes targeting P5/P7 sequences. After equimolar pooling of samples, DNA was concentrated using Agencourt AMPure XP magnetic beads. Libraries were then quantified (absolute quantification) using KAPA^®^ Library Quantification Kits (Roche, Rosny‐sous‐Bois, France) following manufacturer's instructions. Libraries are then loaded on flow cells and processed on the MiSeq^®^ System (Illumina) according to manufacturer's instructions. Sequencing was paired‐end in all cases.

### 2.6 Sequencing data analysis

The downstream data analyses were performed on the Illumina FASTQ files generated by the illumina miseq reporter software (version 2.5.1; Illumina). The primer sequences were removed using fastx_trimmer function from the fastx Toolkit (v0.0.14). The reads were aligned on the genome assembly GRCh37 (hg19) using bwa version 0.7.5a, and bam files were generated using samtools v1.3 (Li *et al.*, 2009; Li and Durbin, 2009). GATK local realignment around known indels and GATK base quality score recalibration was used to recalibrate the reads in BAM files according to the GATK best practices (version 3.5). After the alignment step, coverage statistics were generated using GATK DepthOfCoverage algorithm restricted to the targeted coding sequences. These statistics were used to evaluate the quality of each sample sequencing. Both snv and indel variants were called using Unified Genotyper with default arguments, except that no downsampling was done. Finally, functional effects were predicted using the Oncotator annotation algorithm, along with the ensembl Variant Effect Predictor (VEP) algorithm using RefSeq sequence database downloaded from NCBI on January 26, 2015, and also Annovar annotation (Ramos *et al.*, 2015; Wang *et al.*, 2010).

### 2.7 Variant classification

To ensure only high‐confidence mutation, the resulting vcf files were filtered according to the criteria in the following order: (a) Low quality: QUAL < 100, Allelic Depth of Alternative variant < 6, Genotype Quality < 99, Variant Frequency < 0.1; (b) Frequent polymorphism: referenced in databases (ExAC version 0.3, 1000 genomes phase 1, version 3 and gnomAD) over 0.1% (1000 Genomes Project Consortium *et al.,* 2012; Lek *et al.*, 2016); (c) Rare polymorphism: referenced in databases under 0.1%; (d) Artefacts: detected in a series of 71 nontumoral frozen tissue samples from hepatocellular carcinoma (HCC) patients; (e) Variant silent: ‘Variant_Classification’ = Silent; (f) Variant in noncoding region: ‘Variant_Classification’ = intron, IGR, 3′UTR, 5′UTR, 5′Flank, 3′Flank; (g) Variant with structural consequence: ‘Variant_Classification’ = nonsense, missense, splice site, inframe deletion, inframe insertion, frameshift deletion, and frameshift insertion.

For missense substitutions, we used Polyphen2 HDIV, SIFT, Mutation Taster, and CADD to obtain the prediction of the functional impact on the protein and only substitutions with a CADD score superior to 20 were conserved.

Variants with structural consequence were tagged M1: damaging variants (nonsense, splice site, inframe deletion and insertion, frameshift deletion and insertion, *de novo* start inframe); M2: missense substitutions predicted as damaging by the three tools; M3: missense substitutions predicted as damaging by two of the three tools. Finally, all the damaging variants and *TERT*_prom hotspot mutation sites were confirmed using the integrative genomics viewer (igv) software (Broad Institute, Cambridge, MA, USA).

### 2.8 Quantitative real‐time PCR analysis

Total RNA (1.5 µg) was reverse transcribed in a final volume of 50 µL using the High Capacity cDNA Reverse Transcription Kit (Thermo Fisher Scientific). Quantitative real‐time PCR (qRT‐PCR) products were performed using TaqMan probes and the high throughput BioMark HD system (Fluidigm, Les Ulis, France) following manufacturer’s instructions. Pre‐amplifications of 6 ng cDNA were performed using PreAmp Master Mix (Fluidigm) with a primers mix combining each primer used in the present study except the *18S* probe due to its very high gene expression level. Expression data (*C*_t_ values) were acquired using the fluidigm Real‐Time PCR Analysis software. The mean of five housekeeping genes (*18S*, *ACTB*, *CLTC*, *GAPDH*, *TBP*) was used for the normalization of expression data (Δ*C*_t_).

### 2.9 Classification in molecular subtypes

To assign each sample to the molecular subtypes of the classification in two clusters (C1 and C2) (de Reynies *et al.*, 2014) or four clusters (C1A, C1B, C2A, and C2B) (Blum *et al.*, 2019), a 9‐gene predictor was developed by the ‘Cartes d'identité des tumeurs’ (CIT) program founded by the French ‘Ligue Contre le Cancer’. This predictor was defined using the qRT‐PCR measurements, obtained on a Fluidigm BioMark HD system based on a selection of 69 genes differentially expressed between these subtypes (moderate *t*‐test for the comparison between the two main subtypes C1 and C2 and between the subtypes intra‐C1 and intra‐C2, with fdr < 0.05, absolute fold change > 1.5, and AUC > 0.8) (Table S3). Gene selection was computed by the varSelRF function of the R‐package varSelRF on the Fluidigm dataset restricted to the 63 samples previously assigned to each subtype of both classification systems (C1/C2 and C1A/C1B/C2A/C2B) (Blum *et al.*, 2019; Diaz‐Uriarte, 2007). In brief, varSelRF minimizes the out‐of‐bag error, by successively eliminating the least important variables from random forests. The number of trees ‘ntree’ was set to 10 000 and ‘ntreeIterat’ to 10 000, and default parameters were used otherwise. This procedure resulted in the following selection: *ADAM19*, *ETS1* and *PDCD1LG2* genes for C1 and C2; *CLDN1*, *DSC3,* and *SLC24A3* for C1A and C1B; *CHL1*, *ECM2*, *PTPN13* for C2A and C2B. The predictor was trained using the restricted dataset (63 samples). The subtype prediction was defined by a majority vote across three algorithms [DLDA, DQDA (R package sma), PAM (R package pamr)] and was applied to the remaining samples of the inserm series. Only tumor samples with tumor content of at least 30% after histologic examination were predicted and only predictions with a synthetized score higher than 60 for a particular subtype were taken into account to prevent misleading classification.

### 2.10 Estimation of E.score and S.score

E.score and S.score were estimated using the Wisp R package (https://cit-bioinfo.github.io/WISP/), also developed by CIT program, on qRT‐PCR data with a signature of 55 genes as detailed elsewhere (Blum *et al.*, 2019). Only samples with a cumulated E.score and S.score higher than 50% were taken into account to ensure sufficient tumor content for correct estimation and scores were rescaled after removing the nontumoral component for association analysis with genetic mutations.

### 2.11 Data and statistical analysis

Mutation frequencies and types were retrieved from the release v87 of COSMIC database (https://cancer.sanger.ac.uk/cosmic) (Tate *et al.*, 2019). Data analysis was performed to separate *TERT*_prom and *TERT* core gene mutations from COSMIC database. Dataset of three others series was used: TCGA (Mesothelioma‐TCGA, PanCancer Atlas), GENIE (GENIE Cohort v4.1‐public) (AACR Project GENIE Consortium, 2017), and Bueno series (Bueno *et al.*, 2016). For TCGA and GENIE series, mutation and clinical data were retrieved on November 11, 2018, from cBioPortal for Cancer Genomics (.https://www.cbioportal.org) (Cerami *et al.*, 2012; Gao *et al.*, 2013). TCGA iCluster subtypes and, mutation and clinical data of Bueno series were retrieved from published manuscript (Bueno *et al.*, 2016; de Reynies *et al.*, 2014; Hmeljak *et al.*, 2018). Copy number alterations or fusion, listed in these series, were not taken into account for comparison with the Inserm series data. Cluster comparison of the different molecular classifications was performed by correlating the centroids of their corresponding meta‐profiles as described (Blum *et al.*, 2019).

Statistical tests were performed using graphpad prism version 6.07 software (GraphPad Software, San Diego, CA, USA) except Fisher's exact tests for contingency tables and univariate and multivariate Cox regression analysis, for which R statistical software was used. Lolliplots were drawn using maftools (Mayakonda *et al.*, 2018).

## 3 Results

### 3.1 Genetic alterations in MPM

**Fig. 1:**
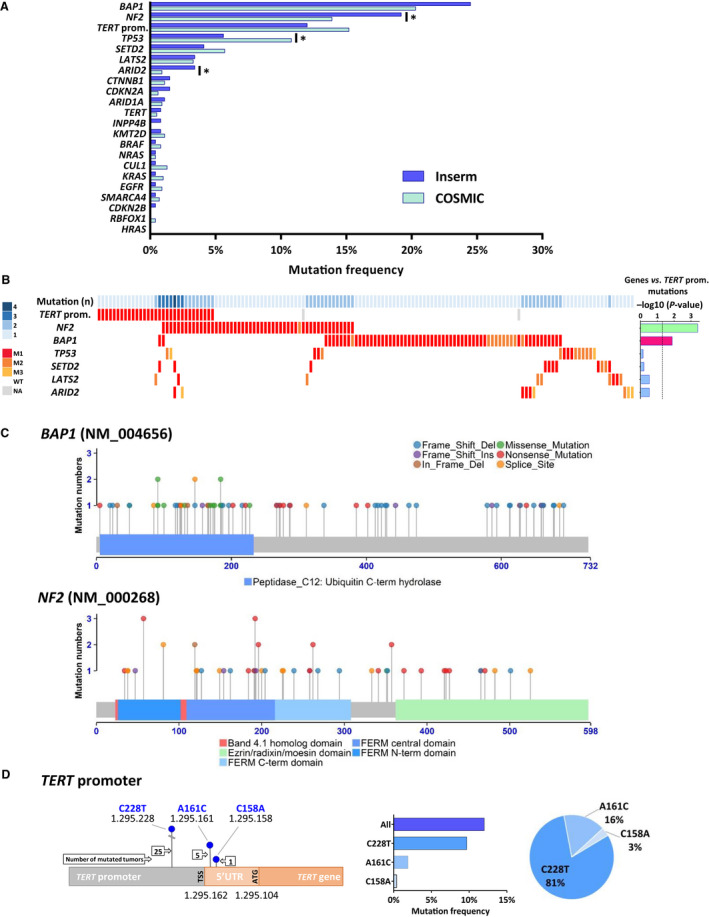
Genetic alterations in MPM. (A) Mutation frequencies in the Inserm and COSMIC series. *P*‐values were determined by Fisher's exact test (*P < 0.05). (B) Distribution of mutations in MPM. MPM tumor samples with at least one mutation in the *TERT* promoter or the six genes most frequently mutated (142 cases) are shown. The number of mutated genes in each sample is indicated by a blue gradient color at the top. Histogram on the right corresponds to −log10 (*P*‐value) of the Fisher's exact test comparing association between *TERT* promoter mutations and other mutations. Lateral bars in magenta and green colors represent significant mutually exclusive and associated mutations, respectively. The black dashed line corresponds to a *P*‐value threshold of 0.05. WT, wild‐type; M, mutated; M1, nonsense substitutions, inframe or frameshift indels and splice sites; M2, missense substitutions damaging; M3, missense substitutions probably damaging. (C) Schematic representation of *BAP1* and *NF2* (Merlin) proteins with mutations mapped (Inserm series). Point mutations are represented as lollipops. Legends of the protein domains and the mutation types are indicated at the bottom and at the top, respectively. (D) Schematic representation of the *TERT* promoter annotated with the localizations of the *TERT* transcription (TSS) and translation (ATG) start sites and the hotspot mutation sites as blue lollipops. Nucleotide numbering indicates the position on chromosome 5 in the GRCh37 assembly. Numbers of mutation at each site are indicated in arrow boxes. On the right, the histogram and the pie chart show the percentage of mutation and the proportions of mutation at each site, respectively.

Mutations in 21 key altered genes in MPM and in the *TERT*_prom were determined by targeted sequencing (see Table S2 for gene selection based on literature data). We identified 200 variants with structural consequences (Table S1; Table S4). Damaging variants were found in 52.3% (139/266) of the MPM tumors (153/266, i.e., 57.5% taking into account mutations in *TERT*_prom), consistent with the percentage of mutated tumors in TCGA series (48.8%), when restricted to the same set of genes with putative driver mutation. Mutations were found in 19 genes, and frequencies are shown in Fig. 1A. The top most frequently mutated genes were *BAP1*, *NF2,* and *TERT*_prom with a mutation frequency of 24.5%, 19.2%, and 12.0%, respectively. The mutation frequencies were similar to those reported in the COSMIC database or other series, except for *TP53* genes (Fig. 1A; Fig. S1).

The distribution of mutations in MPM is shown in the heat map for the six most frequently mutated genes with more than 3% of mutation and *TERT*_prom (Fig. 1B). Up to four of these key genes were mutated in a given tumor. A significant co‐occurrence of mutations in *TERT*_prom and *NF2* was found (*P = *0.0004); that is,* NF2* mutations were more frequent in *TERT*_prom mutant MPM than in wild‐type MPM, 45.2% and 15.8%, respectively. Mutually exclusive mutations were found between *BAP1* and *TERT*_prom (*P = *0.013). Mutations in *BAP1* and *TP53* seem also to be mutually exclusive (not significant) with only one tumor case with both mutated genes. Interestingly, mutations in genes of the SWI/SNF family (*ARID1A*, *ARID2,* and *SMARCA4*) and genes related to histone methylation (*KMT2D*, *SETD2*) were also mutually exclusive. This was not the case for genes belonging to Hippo signaling pathway (*NF2* and *LATS2*), with two MPM showing mutations in both genes, in agreement with our previous observation in MPM primary cell lines (Tranchant *et al.*, 2017).

We mapped the variants on schematic representations of the protein for the six most frequently mutated genes (Fig. 1C; Fig. S2). The proportion of mutation types in Inserm series was also compared to those of COSMIC data (Fig. S3). The mutation profiles have typical tumor suppressor gene (TSG) profile. They are enriched in truncating mutations dispersed randomly all along the gene with specificities according to the genes. *BAP1* and *SETD2* showed a high proportion of frameshift deletions. *NF2* showed a majority of nonsense mutations, but very few missense substitutions contrary to the other genes such as *BAP1* or *TP53* that show a substantial proportion of missense substitutions in their functional domains. Our data were consistent with COSMIC in MPM, but were significantly different from COSMIC pan‐cancer data for *BAP1* and *NF2* (*P* = 0.008 and *P* = 0.004, respectively), suggesting specific mutation types for both genes in MPM.

Three different mutations were identified in the promoter of *TERT*, one in the core promoter (9.7%), that was also the most frequently found in cancers, and two in the 5′‐untranslated region (UTR) of *TERT* mRNA (1.9% and 0.4%) (Fig. 1D). We verified that these mutations were associated with overexpression of *TERT* mRNA as previously described (Tallet *et al.*, 2014). As expected, MPM with *TERT*_prom mutation including mutations in the *TERT* 5′UTR showed a higher expression of *TERT* mRNA than wild‐type MPM (*P* = 0.0015) (Fig. S4A).

### 3.2 Associations between mutation profile and MPM heterogeneity

**Fig. 2:**
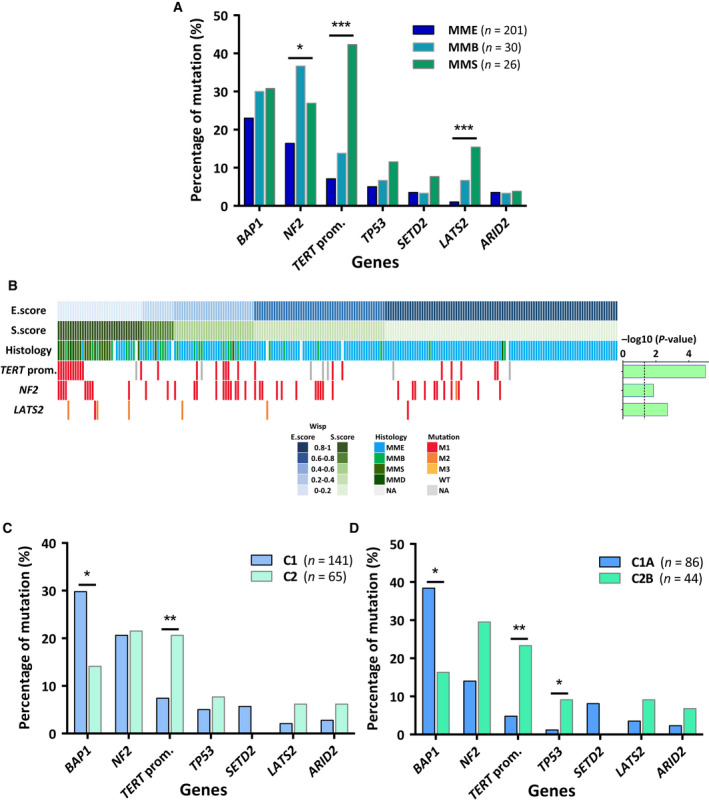
Associations between mutation profile and heterogeneity at the histologic and molecular levels. (A) Associations between mutation profile and histologic types. MMS and MMD were classified together. (B) Heat map of mutation profile in tumor samples along the E.score and S.score (*n* = 231). Distribution of mutations is shown only for genes, which are characterized by a significant association with the E.score or the S.score. Histogram on the right corresponds to −log10 (*P*‐value) of the Student's *t*‐test comparing for a specific gene the E.score or the S.score between MPM with or without any alterations. The black dashed line corresponds to a *P*‐value threshold of 0.05. (C) Associations between mutation profile and transcriptomic subtypes C1 and C2. (D) Associations between mutation profile and transcriptomic subtypes C1A and C2B. *P*‐values were determined by the Fisher's exact tests (*P < 0.05; **P < 0.01; ***P < 0.001) (A, C, and D). WT, wild‐type; M, mutated; M1, nonsense substitutions, inframe or frameshift indels and splice sites; M2, missense substitutions damaging; M3, missense substitutions probably damaging.

Malignant pleural mesothelioma heterogeneity was characterized at the histologic and molecular levels in Inserm series. First, we focused on the link between the mutation profile, considering the six most frequently mutated genes and *TERT*_prom, and the main three MPM histologic types, that is, MME, MMB, and MMS. Mutations in *TERT_prom*, *NF2,* and *LATS2* were significantly less frequent in MME than in non_MME samples (MMB and MMS) (Fig. 2A; Fig. S5A). Associations between *NF2* and *LATS2* mutation profiles and histologic types were confirmed by analyzing three other MPM series: Bueno, TCGA, and GENIE series (Fig. S5B–F).

Recently, we described a new method to take into account MPM heterogeneity based on a deconvolution approach on gene expression, which allows to define epithelioid‐like and sarcomatoid‐like components and to determine their proportions, the E.score and the S.score, respectively, in a given tumor sample (Blum *et al.*, 2019). These scores were determined in Inserm series (Table S1), and their associations with the mutational profile were investigated. Significant positive associations were found between the S.score and *TERT_prom*, *NF2,* and *LATS2* mutations, the same mutations associated with the non_MME histologic subtypes (Fig. 2B). The E.score and the S.score were also estimated for the MPM samples of Bueno and TCGA series (Blum *et al.*, 2019). Analysis of *NF2* and *LATS2* mutation data showed also significant statistical associations in these series (Fig. S6).

In a previous publication, we defined a molecular classification of MPM in two subtypes C1 and C2 (de Reynies *et al.*, 2014). More recently, we also defined intra‐subtypes (C1A, C1B, C2A, and C2B) by subdividing C1 and C2 into two groups (Blum *et al.*, 2019). We compared these intra‐subtypes with subtypes already published (Bueno *et al.*, 2016; Hmeljak *et al.*, 2018) and those defined by unsupervised clustering from other public series (Lopez and Gordon series) (Blum *et al.*, 2019) by correlating the centroids of their corresponding meta‐profiles (Fig. S7A). The correlation matrix showed two main groups of highly correlated clusters present in all datasets, which contained the C1A subtype associated with Epithelioid and iCluster 1 subtypes and the C2B subtype associated with Sarcomatoid and iCluster 4 subtypes from Bueno and TCGA series, respectively. Tumor samples of Inserm series were predicted in 2 and 4 subtypes molecular classification using a predictor based on nine genes: *ADAM19, ETS1,* and *PDCD1LG2* for C1 and C2; *CLDN1*, *DSC3,* and *SLC24A3* for C1A and C1B; *CHL1*, *ECM2*, and *PTPN13* for C2A and C2B subtype prediction (Fig. S8). We showed a significant enrichment of *BAP1* mutations in the C1 subtype, and of *TERT*_prom in the C2 subtype (Fig. 2C). Distribution of mutations between intra‐subtypes (C1A, C1B, C2A, and C2B) is shown in Fig. S7B. Comparison of mutation profiles between C1A and C2B subtypes highlighted increase frequency of *BAP1* and *SETD2* mutations in C1A subtype and, *TERT*_prom, *NF2*, *TP53*, *ARID2,* and *LATS2* mutations in C2B subtypes (Fig. 2D). These associations were only significant for *BAP1*, *TP53,* and *TERT*_prom in Inserm series, but pan‐series analysis showed that these associations were all significant except for *LATS2* and *ARID2* (Fig. S7C–I).

### 3.3 Associations between mutation profile and clinical and epidemiological data

**Fig. 3:**
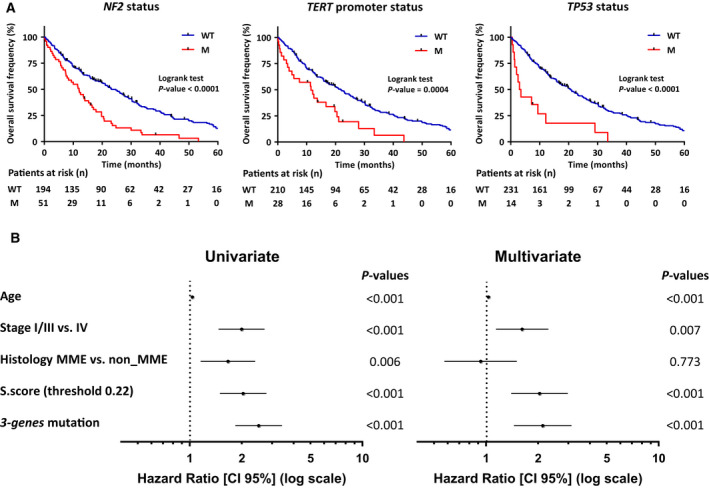
Associations between mutation profile and overall survival. (A) Kaplan–Meier plots of overall survival in patients with wild‐type (blue curve) or mutated (red curve) *NF2*, *TERT* promoter, and *TP53*. *P*‐values were determined by the Log‐rank tests. (B) Univariate and multivariate Cox regression analysis of overall survival in MPM patients. Forest plots show hazard ratios (HR) and 95% confidence interval (CI) for overall survival according to age at diagnostic, tumor stage, histology, and S.score based on a threshold of 0.22 and mutation status. For histology, MMB, MMS, and MMD were classified as non_MME. For mutation status, samples were discriminated for the presence or the absence of at least one mutation in one of the genes *TP53* or *NF2*, or in the *TERT* promoter (*3‐gene* mutation). *P*‐values of the Wald test for all variables are indicated at the right of each forest plot. WT, wild‐type; M, mutated.

We did not find a significant association between mutation profile and age, gender or tobacco consumption. *NF2* mutations were significantly associated with asbestos exposure status (*P* = 0.036) and were more frequent in nonexposed patients (31%) than in exposed patients (17%) (Fig. S9A). *TERT*_prom and *NF2* mutations were significantly associated with the tumor stage (*P* = 0.025 and *P* = 0.007, respectively) and showed significant higher mutation rate in patients with stage IV tumors (20% and 28%, respectively) than in patients with stage I/III tumors (9% and 13%, respectively) (Fig. S9B). Strong significant associations were observed with overall survival. Overall survival frequency was lower in patients with MPM mutated for *TERT*_prom, *TP53,* and *NF2* compared to patients with MPM wild‐type (Fig. 3A). These associations are also found when considering only MME samples and non_MME samples except for *TP53* in non_MME samples (Fig. S10). Multivariate analysis considering age at diagnostic, tumor stage, histology, S.score based on a threshold of 0.22, which was shown to be the more predictive for prognosis (Blum *et al.*, 2019), and mutation status in these three genes showed that the mutation status is predictive of prognosis only for *TP53* and not for *TERT*_prom and *NF2* (Fig. S11). However, multivariate analysis considering MPM samples with at least one mutation in *TP53*, *NF2,* or *TERT*_prom highlighted the strong prognosis value of all three genes considered together (Fig. 3B).

### 3.4 Mutations profile between samples from the same patient

**Fig. 4:**
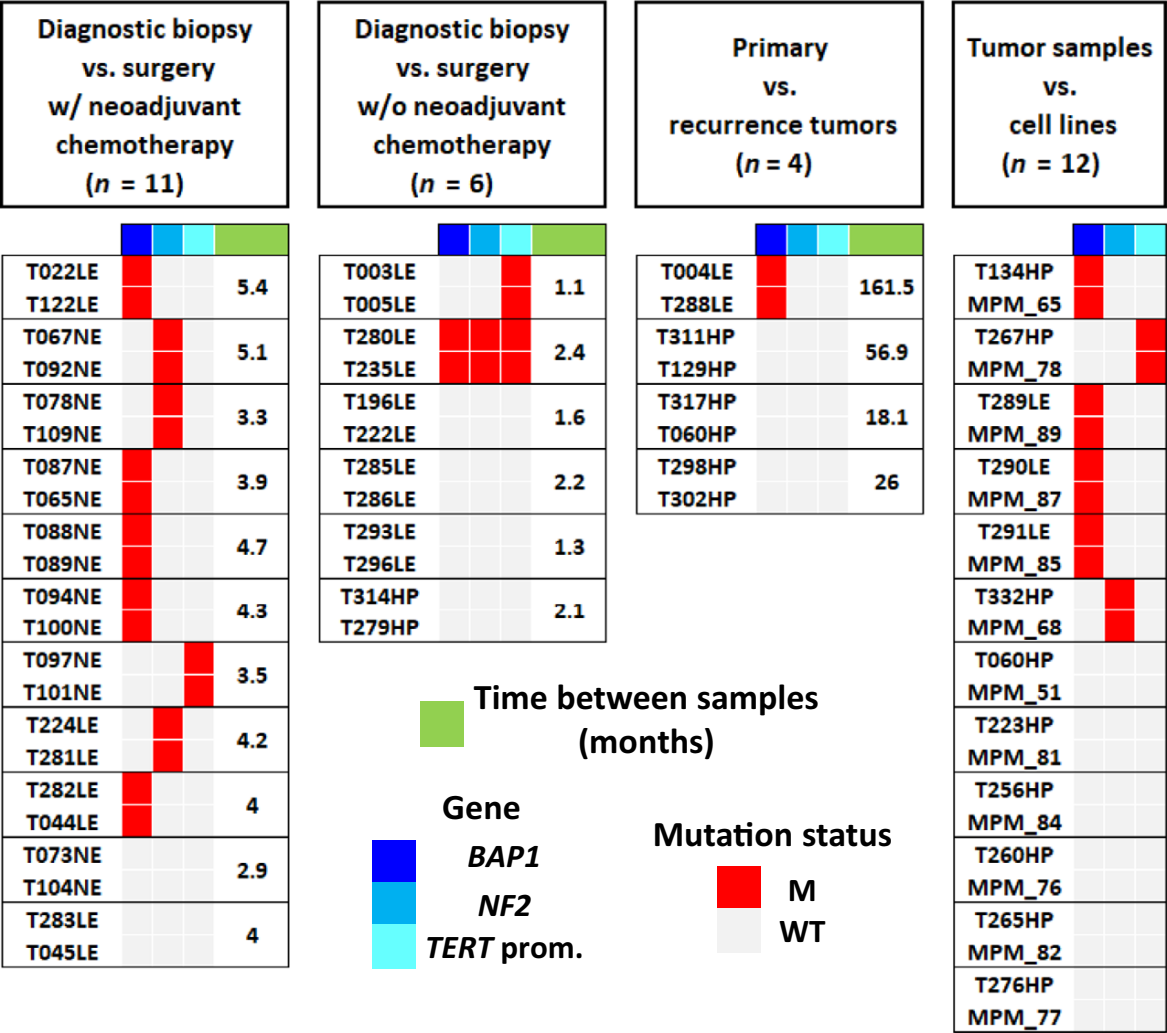
Mutation profile of tumor samples from the same patient. Heat map shows the genetic alterations identified in tumor samples collected from the same patient. Frozen tumor samples were collected either from diagnostic biopsy or surgery resection, with or without neo‐adjuvant chemotherapy, and from primary and recurrence tumors. Cell lines were also established from MPM and compared to frozen tumor samples. Legends are indicated at the bottom. *n*, number of tumor sample pairs; WT, wild‐type; M, mutated.

Our MPM collection includes several different tumor samples including diagnostic biopsies, surgery resections, and tumor samples of recurrence from the same patient: (a) Eleven pairs of tumor samples corresponding to diagnostic biopsies and surgery resections, with neo‐adjuvant therapy, collected with a spacing of 2.9–5.4 months. (b) Six pairs of samples corresponding to diagnostic biopsies and surgery resections, without neo‐adjuvant therapy, collected with spacing of 1.1–2.4 months. (c) Four pairs of tumor samples corresponding to primary tumors and recurrence tumors, with a spacing of 18.1–161.5 months (Table S5). Mutations were identified in 12 sample pairs, and there was no difference between mutational statuses between both samples in all the cases. We also established 12 MPM primary cell lines from tumor samples included in the Inserm series. Among six sample pairs with characterized mutations, mutations were identical between tumor samples and cell lines (Fig. 4).

## 4 Discussion

In previous studies, we defined a transcriptomic molecular classification of MPM and investigated inter‐ and intratumor heterogeneity using a deconvolution approach (Blum *et al.*, 2019; de Reynies *et al.*, 2014). The present study characterizes the genetic alterations in the most frequently mutated genes in MPM in a large series of 266 frozen tumors well annotated for clinical and histologic and molecular heterogeneity.

Inserm series was one of the largest series with the one of Bueno *et al. *(2016) used so far to screen mutations in key altered genes in MPM, and the largest for *TERT*_prom mutations that are not evaluated by exome sequencing. In the Inserm series, *TERT*_prom is the third locus most affected by mutations in MPM and deserved to be considered in the genetic landscape of MPM given the importance of telomerase upregulation in cancer (Pestana *et al.*, 2017). The present study allows also drawing up an accurate assessment of the frequency of gene mutations in a homogeneous series of MPM. We highlight genes such as *ARID2*, only mentioned as mutated in a previous study on MPM cell lines (Yoshikawa *et al.*, 2015). At the opposite, we show that genes that have been suggested as frequently mutated in small series of MPM are in fact only rarely mutated, such as *CUL1* (Guo *et al.*, 2015). Even if the large majority of the mutations identified in this study are likely somatic mutations, we cannot exclude that some of the mutations correspond to germline mutations especially in *BAP1* gene, the gene with the highest frequency of germline mutations in MPM (Panou *et al.*, 2018; Pastorino *et al.*, 2018). Among the 65 patients showing *BAP1* mutation of Inserm series (Table S1), none had previous cutaneous or uveal melanoma, renal cell carcinoma, basal cell carcinoma, meningioma, or cholangiocarcinoma, which were recently described in a study on 181 families carrying *BAP1* germline variants as the core tumor spectrum for the *BAP*1 tumor predisposition syndrome (Walpole *et al.*, 2018).

Mutation frequencies are consistent between Inserm series and other series. The only significant discrepancy was for *TP53* mutation frequencies suggesting a variability between MPM series for this gene (Fig. S1). Of note, this study did not take into account large exon deletions as the targeted sequencing does not allow detecting accurately large deletions even based on the sequencing depth of coverage. This does not have an impact on *TP53* or *TERT*_prom alterations frequencies, but those of *CDKN2A*, *BAP1,* and *NF2* are most likely underevaluated as it is the case in most NGS studies due to the contamination of tumor samples by normal cells. For *NF2*, we verified that taking into account large deletions did not change the association between *NF2* mutation status and, histologic and molecular subtypes or gradients (Fig. S12).

Our detailed mutation analysis also gives a precise overview of the mutation types profile observed in the main TSG mutated in MPM and in the *TERT*_prom. *BAP1* and *SETD2* are enriched in deletion consistent with their chromosome localizations in 3p21 region, which harbors multiple noncontiguous minute deletions in MPM (Yoshikawa *et al.*, 2016). *NF2* shows few missense mutations but several mutations leading to the production of truncated protein forms, consistent with its role as a multifunctional protein interacting with several partners through different parts of the protein (Sato and Sekido, 2018). As for *BAP1* and *NF2*, *TERT*_prom mutation types present characteristics specific to MPM. The three mutations in *TERT*_prom were previously described in other cancers and generate *de novo* ETS binding sites (Huang *et al.*, 2015). However, while the C228T mutation is the most frequent in MPM as in other tumors, the C250T, the second most common mutation in tumors, is not found in MPM. The two others less frequent mutation sites A161C and C1581 in MPM are mostly found in the bladder transitional carcinoma in COSMIC database. We also validated the correlation between promoter mutation and *TERT* overexpression in MPM, observed previously (Tallet *et al.*, 2014). Interestingly, literature data suggest that *BAP1* could downregulate *TERT* expression (Linne *et al.*, 2017). As we found mutually exclusive mutations between both alterations, we compared *TERT* expression based on the *BAP1* mutation status, but did not find any significant association in MPM (Fig. S4b).

Most of the mutated genes in MPM are TSG and untargetable gene directly. However, we identified mutations in genes known as being targetable genes (Table S3). For example, the mutation in the oncogene *KRAS* Q61H has been referenced in COSMIC database (COSM555) and is a hotspot known to be oncogenic and found in several other malignancies such as large intestine and lung carcinomas. Preclinical and preliminary clinical data suggest that cancers with *KRAS* mutant may be sensitive to MEK or ERK inhibitors (Sullivan *et al.*, 2018). The *NRAS* Q61K mutation is also an oncogenic hotspot (COSM580), and there is promising clinical data in patients with oncogenic *NRAS*‐mutant melanoma treated with the MEK1/2‐inhibitor, binimetinib (Dummer *et al.*, 2017). Unfortunately, this concerns only a very small subset of MPM patients. For most patients, genes or pathways deregulated as a consequence of TSG inactivation should be targeted, as it was suggested for hippo signal pathway linked to *NF2* and *LATS2* inactivation (Felley‐Bosco and Stahel, 2014; Sato and Sekido, 2018).

**Fig. 5:**
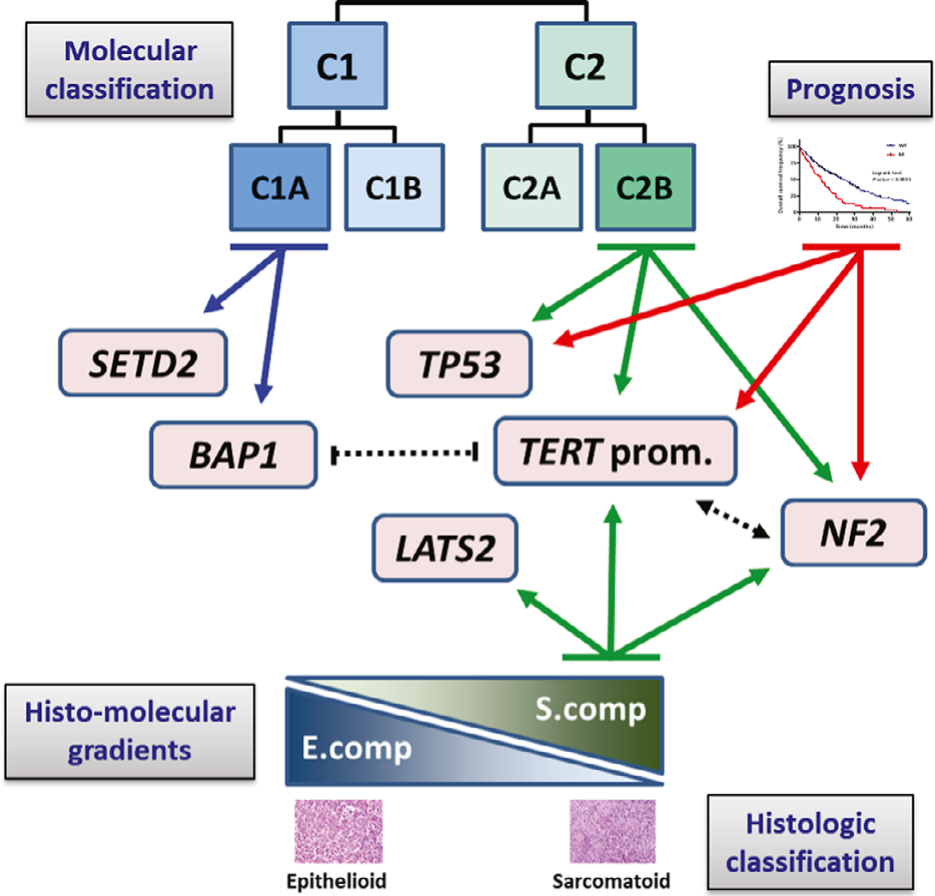
Schematic representation of the link between the genetic landscape and tumor heterogeneity in MPM. Solid lines with arrows indicate significant associations between mutated genes and histologic or molecular classifications, histo‐molecular gradients or prognosis. Dotted lines with arrows or dashes indicate significant association or exclusion between mutated genes, respectively.

The key input of this study is to precise the genetic landscape taking into account MPM heterogeneity. We identified significant increase in the mutation rate for *LATS2, NF2,* and *TERT*_prom in non_MME allowing us to confirm in a larger MPM series the association with *TERT*_prom previously observed (Tallet *et al.*, 2014) and to demonstrate at the statistical level the previously suggested association with *NF2* (Sato and Sekido, 2018). Mutations in *LATS2*, *NF2,* and *TERT*_prom were also positively associated with the S.score, bringing new insights on the intratumor heterogeneity and strengthening the link between these mutations and the sarcomatoid cell type. *NF2* and *TP53* mutations were previously found to be associated with the S.score, based on TCGA series data (Blum *et al.*, 2019). Here, we confirmed the association of *NF2* mutations in this larger Inserm series, possibly due to the variability of *TP53* mutations between MPM series mentioned above. Another important point concerns the occurrence of gene mutations according to the molecular classifications in subtypes. We confirmed the previously described association of *BAP1* mutations to the C1 subtype (de Reynies *et al.*, 2014) and identified a significant enrichment of *TERT*_prom mutations in the C2 subtype. Further classifications in four subtypes were also proposed in several studies (Blum *et al.*, 2019; Bueno *et al.*, 2016; Hmeljak *et al.*, 2018). Here, we focused on the extreme subtypes, that is, the C1A/Epithelioid/iCluster 1 and the C2B/Sarcomatoid/iCluster 4 subtypes, as they are detected in all MPM series. Hmeljak *et al.* (2018) reported a strong significant association of *BAP1* mutation with iCluster 1 and an enrichment of *LATS2* mutation in iCluster 4. We also observed the same associations in Inserm series. Moreover, we highlighted new significant associations between *TERT_prom* and *TP53* mutations, and C2B subtype. Pan‐series analysis confirmed all these associations and revealed a significant association of *NF2* mutations with C2B subtype. Interestingly, *TERT*_prom and *NF2* mutations are associated with histologic and molecular classifications, and molecular gradients, but not *TP53* and *BAP1* mutations (Fig. 5). These results highlight the complexity of MPM heterogeneity and suggest that classification in subtypes even if related to histologic types take into account another degree of heterogeneity. *BAP1‐* and *TP53*‐mutated tumors may form specific subtypes inside epithelioid and sarcomatoid enriched tumors, respectively. In a previous study, we demonstrate the impact of epigenetic mechanisms in the establishment of epithelioid and sarcomatoid‐related cell entities (Blum *et al.*, 2019). Altogether, our new results also highlight the contribution of different genetic‐related mechanism and support different ways for mesothelial cell neoplastic transformation.

Previous studies reported an association between loss of specific chromosome regions and asbestos exposure (Borczuk *et al.*, 2016; Jean *et al.*, 2011). However, to our knowledge, our study is the first to identify a link between gene mutations and asbestos status. In our series, *NF2* mutation was the most frequent alteration in asbestos nonexposed patients with a third of patients carrying *NF2* mutation. Only one study reported a MPM patient with constitutional *NF2* mutation missense mutation (Baser *et al.*, 2005). Furthermore, in two recent studies screening germline cancer susceptibility mutations in large cohort of MPM patients, *NF2,* was not identified as a cancer susceptibility gene (Panou *et al.*, 2018; Pastorino *et al.*, 2018), suggesting that *NF2* mutations observed in our series in unexposed patient are likely somatic. This high frequency of mutations supports *NF2* as a key driver of asbestos independent mesothelial carcinogenesis that was previously suggested in mice models. The development of peritoneal mesothelioma was observed in genetically engineered mice heterozygous in *Nf2* without asbestos exposure (Giovannini *et al.*, 2000). Conditional mouse model leading to both *Ink4a/Arf* and *Nf2* inactivation was shown to develop malignant thoracic mesothelioma at a high incidence without asbestos exposure, mostly of sarcomatoid type (Jongsma *et al.*, 2008). Interestingly, *LATS2* is the second gene showing the mutations more frequent in nonexposed patients (7%) than in exposed patients (2%). Both *NF2* and *LATS2* belong to the hippo signal pathway known to be crucial for asbestos‐driven carcinogenesis and, based on our data, also for asbestos‐independent mesothelial carcinogenesis.

One of the major strengths of our study is to demonstrate the strong link between the mutation status of *TERT*_prom, *NF2* and *TP53*, and overall survival. Accordingly, *TERT*_prom and *NF2* mutations were significantly more frequent in MPM with an advanced stage. The prognosis interest based on the mutational status was already reported for *TP53* by Bueno *et al. *(2016), but not for other genes. Of note, in agreement with the *NF2* mutations prognosis value, an immunohistochemistry study reported that low merlin expression is an indicator of poor prognosis in MPM patients (Meerang *et al.*, 2016). *TERT*_prom mutation has been associated with worse prognosis in some cancers including meningioma but not in MPM (Lu *et al.*, 2019). Multivariate analysis confirms the prognosis value of the S.score and highlights the prognosis value of the three genes together that could be an alternative for evaluating the prognosis in clinic.

The rapid evolution of MPM is challenging for targeted therapy. The comparison of mutation profile of tumor samples collected at different time points from a same patient did not show any difference. One tumor sample pair (T004LE and T288LE), corresponding to primary versus recurrence tumors, showed two *BAP1* mutations (K337fs and N157fs) present in both samples. It is impressive to find the same mutation since the two samples were collected within a 13‐year interval of time. We cannot completely exclude a germline mutation in *BAP1*, but to our knowledge two co‐occurring *BAP1* germline mutations were not previously identified in a same family. Obviously, this stability over time of the mutational profile should be confirmed in larger series. As MPM cell lines are useful for studying mesothelial carcinogenesis and for identifying new therapies by testing anticancer drugs, we also characterized the mutations in primary cell lines established from sequenced tumor samples. Our results show that mutations in primary cell lines are representative from the mutations present in the tumor of the patient.

## 5 Conclusion

Overall, the present study provides a comprehensive overview of the genetic landscape of MPM taking into account the histologic and molecular heterogeneities. This better understanding of heterogeneity at the genetic level should facilitate the implementation of strategies to develop precision medicine for MPM, which is crucial for this incurable cancer. Our findings also highlight the strong prognostic value of genetic alterations relevant for clinical application.

## Conflict of interest

The authors declare no conflict of interest.

## Author contributions

CM, JZ‐R, M‐CJ, and DJ are responsible for the study concept and design. VH, PH, M‐CC, EP, AS, IM, LG, CB, and FLP‐B provided patient materials. VH, PH, FM, JW, and J‐BA collected and organized clinical data. VH, M‐CC, LG, and CB performed histologic analysis of tumor samples. LQ, CM, RT, JW, FM, and J‐BA contributed to sample preparation and quality control. LQ and CM performed the acquisition of genetic data. SC, YB, CM, and SI developed the bioinformatics tools and the pipeline analysis. YB, CM, LQ, and DJ performed the analysis and interpretation of data. CM, LQ, M‐CJ, and DJ were major contributors in writing the manuscript. FLP‐B, JZ‐R, M‐CJ, and DJ are responsible for the study supervision. All authors read and approved the final manuscript.

## Supporting information


**Fig. S1.** Mutation frequencies in four MPM series.
**Fig. S2.** Genetic alterations in MPM.
**Fig. S3.** Proportions of different mutation types in MPM.
**Fig. S4.** Gene expression of *TERT* gene in MPM.
**Fig. S5.** Associations between mutation profile and histologic types.
**Fig. S6.** Associations between mutation profile and molecular gradients.
**Fig. S7.** Associations between mutation profile and molecular subtypes.
**Fig. S8.** Gene expression of the 9‐gene predictor.
**Fig. S9.** Associations between mutation profile and asbestos exposure status and tumor stage.
**Fig. S10.** Associations between mutation profile and overall survival.
**Fig. S11.** Prognostic value of *NF2*, *TERT* promoter and *TP53 *mutations.
**Fig. S12.** Associations between *NF2* mutation status including large deep deletions and, histologic and molecular subtypes or gradients.

**Table S1.** Clinical and molecular annotations of MPM samples (Inserm series).

**Table S2.** Genes of the targeted sequencing.

**Table S3.** Deregulated genes between molecular subtypes (Table S3A) and associations to the different subtypes (Tables S3B‐D).

**Table S4.** Variants with structural consequences in genes and variants in *TERT* promoter identified by targeted sequencing.

**Table S5.** Tumor samples from the same patient.
