# Supplementary material for: Genetic alterations of malignant pleural mesothelioma: association with tumor heterogeneity and overall survival
Source: Mol Oncol. 2020 Mar 10;14(6):1207–23. doi: 10.1002/1878-0261.12651 (PMC7266286; doi:10.1002/1878-0261.12651)
Supplement: Supplementary file 1 — Fig. S1. Mutation frequencies in four MPM series. Fig. S2. Genetic alterations in MPM. Fig. S3. Proportions of different mutation types in MPM. Fig. S4. Gene expression of TERT gene in MPM. Fig. S5. Associations between mutation profile and histologic types. Fig. S6. Associations between mutation profile and molecular gradients. Fig. S7. Associations between mutation profile and molecular subtypes. Fig. S8. Gene expression of the 9‐gene predictor. Fig. S9. Associations between mutation profile and asbestos exposure status and tumor stage. Fig. S10. Associations between mutation profile and overall survival. Fig. S11. Prognostic value of NF2, TERT promoter and TP53 mutations. Fig. S12. Associations between NF2 mutation status including large deep deletions and, histologic and molecular subtypes or gradients. [file MOL2-14-1207-s001.pdf]

Supplementary Figures

Figure S1. Mutation frequencies in four MPM series.

Figure S2. Genetic alterations in MPM.

Figure S3. Proportions of different mutation types in MPM.

Figure S4. Gene expression of *TERT* gene in MPM.

Figure S5. Associations between mutation profile and histologic types.

Figure S6. Associations between mutation profile and molecular gradients.

Figure S7. Associations between mutation profile and molecular subtypes.

Figure S8. Gene expression of the 9-gene predictor.

Figure S9. Associations between mutation profile and asbestos exposure status and tumor stage.

Figure S10. Associations between mutation profile and overall survival.

Figure S11. Prognostic value of *NF2*, *TERT* promoter and *TP53* mutations.

Figure S12. Associations between *NF2* mutation status including large deep deletions and, histologic and molecular subtypes or gradients.

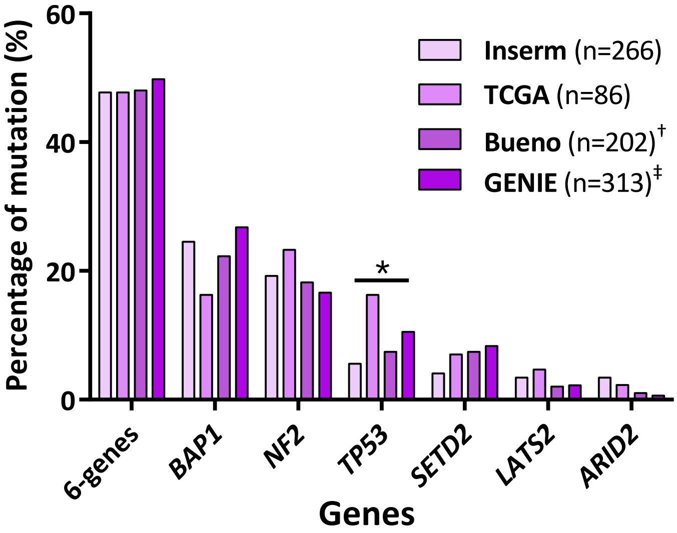

Figure S1 Mutation frequencies in four MPM series.
Mutation frequencies of MPM tumor samples with mutations in *BAP1*, *NF2*, *TP53*, *SETD2*, *LATS2* or *ARID2* genes or with at least one mutation in one of these six genes (6-genes) are shown in Inserm, Bueno, TCGA and GENIE series. P-values were determined by the Fisher's exact test (*: p<0.05; **: p<0.01; ***: p<0.001). n: number of tumor samples; † the number of sequenced tumor samples was 99 for *LATS2* and *ARID2*; ‡ the number of sequenced tumor samples was 139 for *LATS2*.

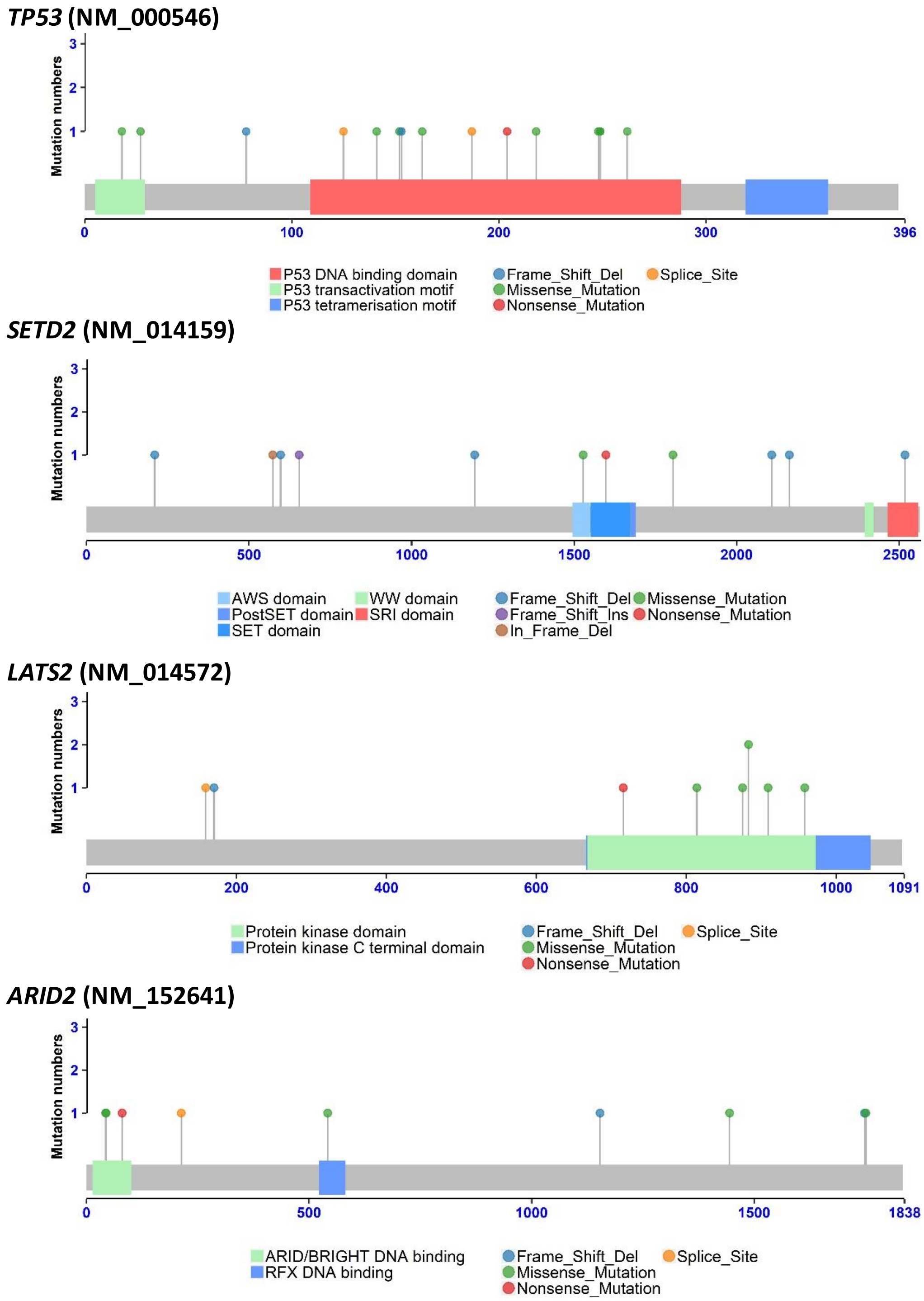

Figure S2 Genetic alterations in MPM.

Schematic representation of P53, SETD2, LATS2 and ARID2 proteins with mutations mapped (Inserm series). Point mutations are represented as lollipops. Legends of the protein domains and the mutation types are indicated at the bottom left and right, respectively.

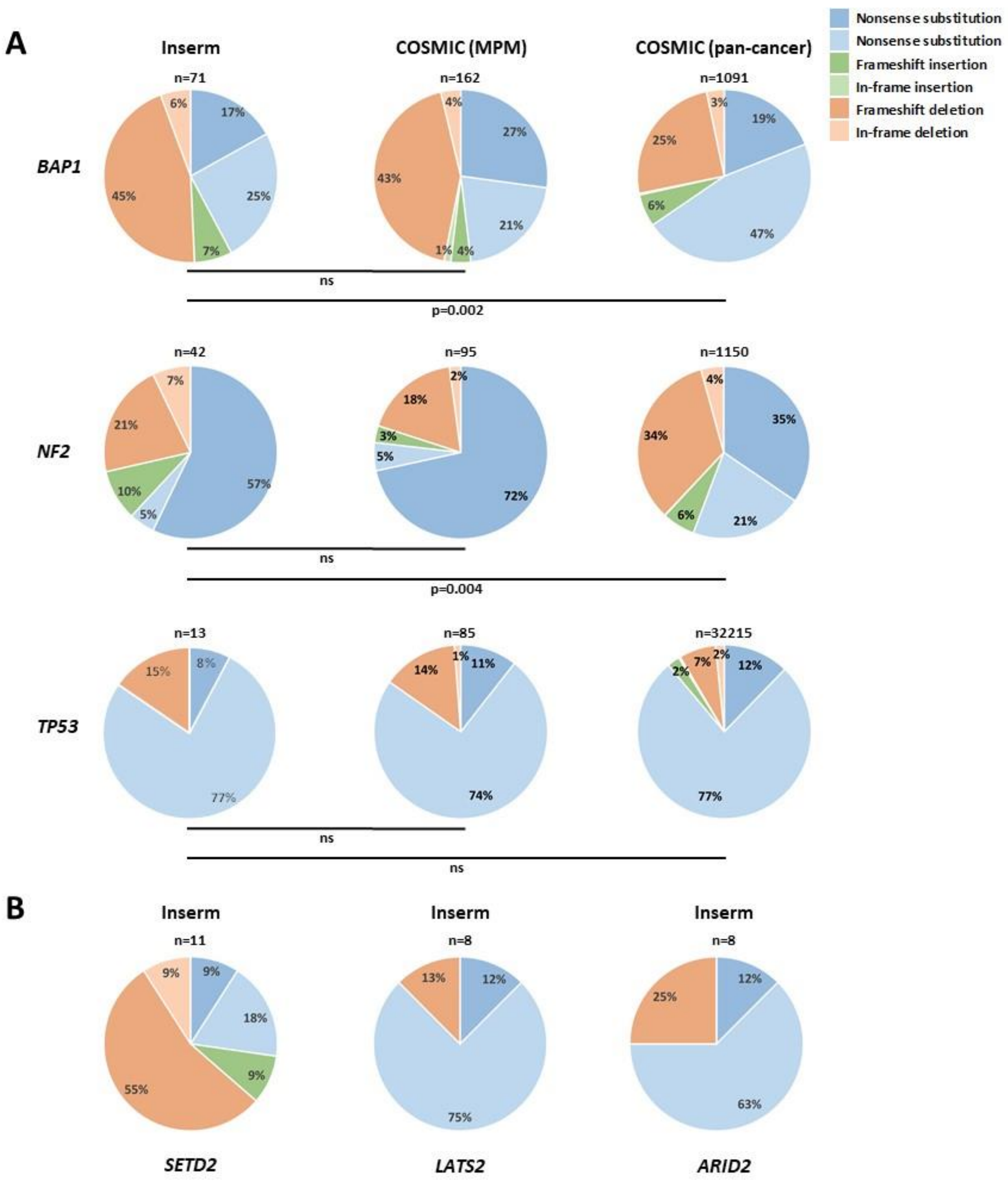

Figure S3 Proportions of different mutation types in MPM.
(A) Proportions of different mutation types in *BAP1*, *NF2* and *TP53* genes in Inserm, MPM COSMIC and pan-cancer COSMIC series. P-values were determined by the Fisher's exact test. (B) Proportions of different mutation types in *SETD2*, *LATS2* and *ARID2* genes in Inserm series. Since there are few variants reported in these genes, we did not compare to COSMIC data. Splice site alterations due to substitutions in intronic region or synonymous substitutions, representing 7.8% in *BAP1*, 23.6% in *NF2*, 13.3% in *TP53*, 0% in *SETD2*, 11.1% in *LATS2* and 11.1% in *ARID2* of all of the variants detected in each gene, were not taken into account in the chart pie because of their absence in the COSMIC database. Legends of the chart pies are indicated at the top left. MPM: malignant pleural mesothelioma; n: number of tumor samples; ns: not significant.

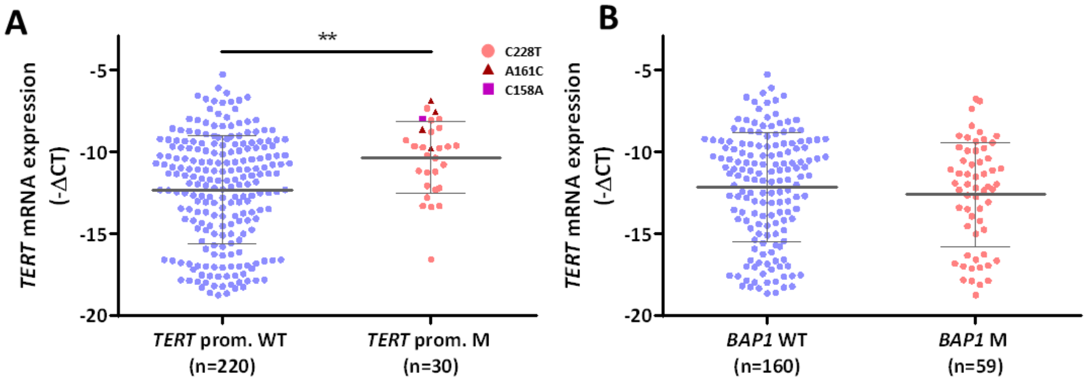

Figure S4 Gene expression of *TERT* gene in MPM.

MRNA expression of *TERT* gene was quantified by RT-qPCR in MPM frozen tumor samples of the Inserm series. (A) Comparison of *TERT* gene expression between *TERT* promoter mutated and wild-type MPM. The mutation sites of *TERT* promoter are highlighted in the dot plot. (B) Comparison of *TERT* gene expression between *BAP1* gene mutated and wild-type MPM in *TERT* promoter wild-type MPM. P-values were determined by the Mann-Whitney test (*: p<0.05; **: p<0.01; ***: p<0.001). WT: wild-type; M: mutated.

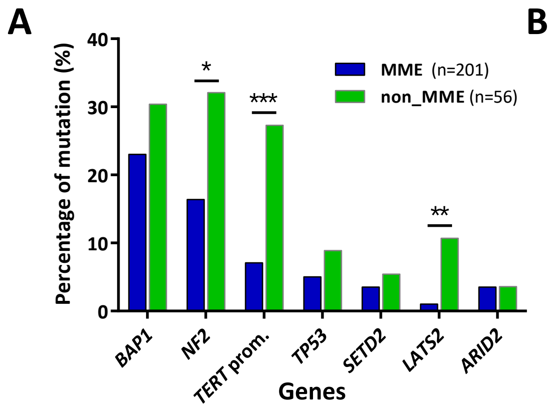

B

Gene	Histology	Inserm	Bueno	TCGA	GENIE	All series
	MME	201	134	61	19	563
NF2	MMB	30	61	23	17	166
	MMS	26	8	2	7	52
	MME	201	70	61	68	400
LATS2	MMB	30	22	23	8	83
	MMS	26	7	2	5	40

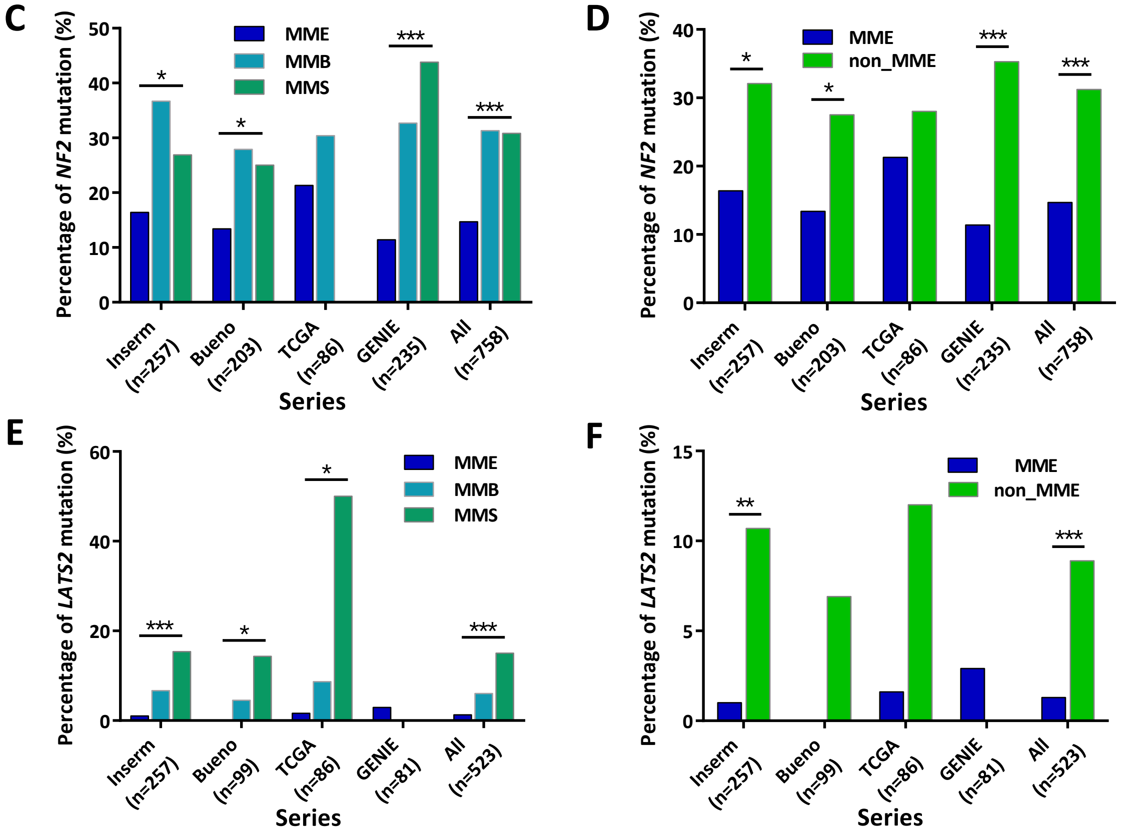

Figure S5 Associations between mutation profile and histologic types.
(A) Associations between mutation profile and, MME or non_MME histologic types in Inserm series. MMB, MMS and MMD were classified as non_MME. (B) The numbers of tumor samples of the different histologic types in each MPM series (Inserm, Bueno, TCGA and GENIE series) and in all series are shown in the table. (C-F) Associations between *NF2* (C and D) and *LATS2* (E and F) mutation status and, MME, MMB and MMS (C and E) or MME and non_MME (D and F) histologic types in each MPM series and in all series. P-values were determined by the Fisher's exact test (*: p<0.05; **: p<0.01; ***: p<0.001). MME: epithelioid MPM; MMB: biphasic MPM; MMS: sarcomatoid MPM; MMD: desmoplastic MPM.

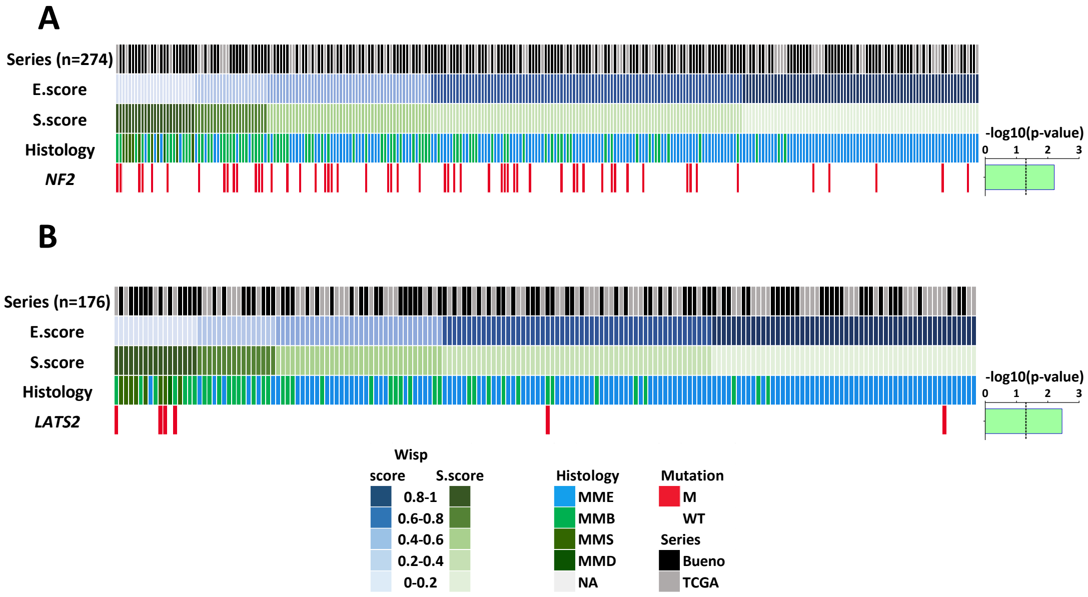

Figure S6 Associations between mutation profile and molecular gradients.
(A and B) Heat maps of the mutation status of *NF2* (A) or *LATS2* (B) genes in tumor samples of TCGA and Bueno series classified according to the E.score and S.score. Histogram on the right corresponds to -log10(p-value) of the Student's t test comparing for a specific gene the E.score or the S.score between MPM with or without any alterations. The black dashed line corresponds to a p-value threshold of 0.05. MME: epithelioid MPM; MMB: biphasic MPM; MMS: sarcomatoid MPM; MMD: desmoplastic MPM; WT: wild type; M: mutated.

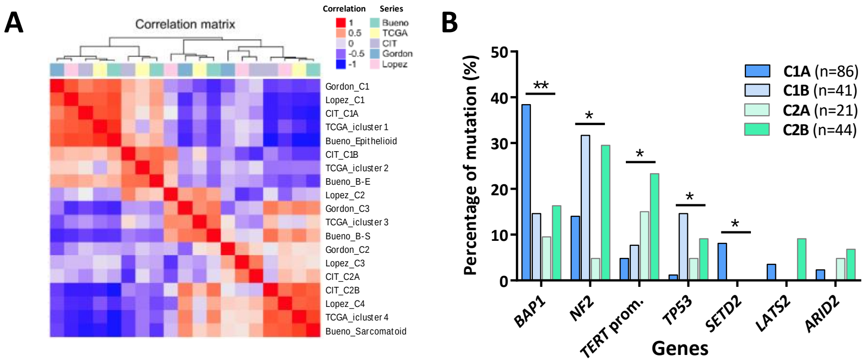

C

Molecular subtypes	Inserm	Bueno	TCGA	All series
C1A / Epithelioid / iCluster 1	86	49	19	154
C2B / Sarcomatoid / iCluster 4	44	29	20	93

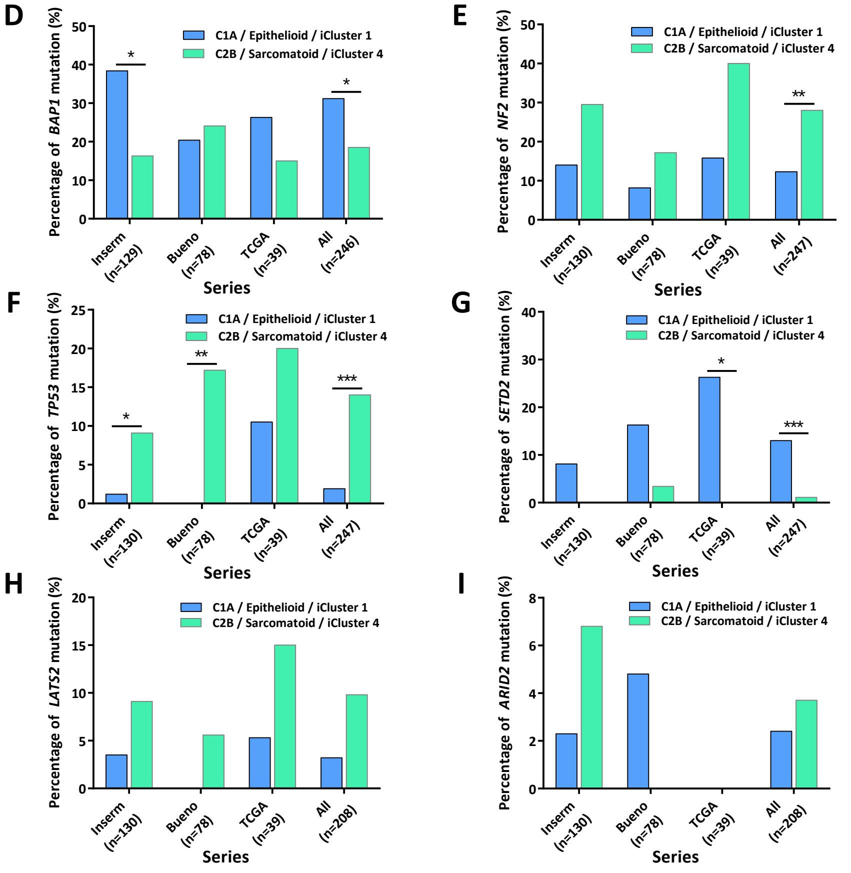

Figure S7 Associations between mutation profile and molecular subtypes.
(A) Correlation matrix of centroid profiles of all subtypes from the different molecular classifications in five MPM series. Legends are indicated at the top right. (B) Associations between mutation profile and transcriptomic subtypes C1A, C1B, C2A and C2B in Inserm series. (C) The numbers of tumor samples of the different molecular subtypes in each MPM series (Inserm, Bueno, and TCGA series) and in all series are shown in the table. (D-I) Associations between *BAP1* (D), *NF2* (E), *TP53* (F), *SETD2* (G), *LATS2* (H) and *ARID2* (I) mutation status and molecular subtypes in each MPM series and in all series. P-values were determined by the Fisher's exact test (*: p<0.05; **: p<0.01; ***: p<0.001).

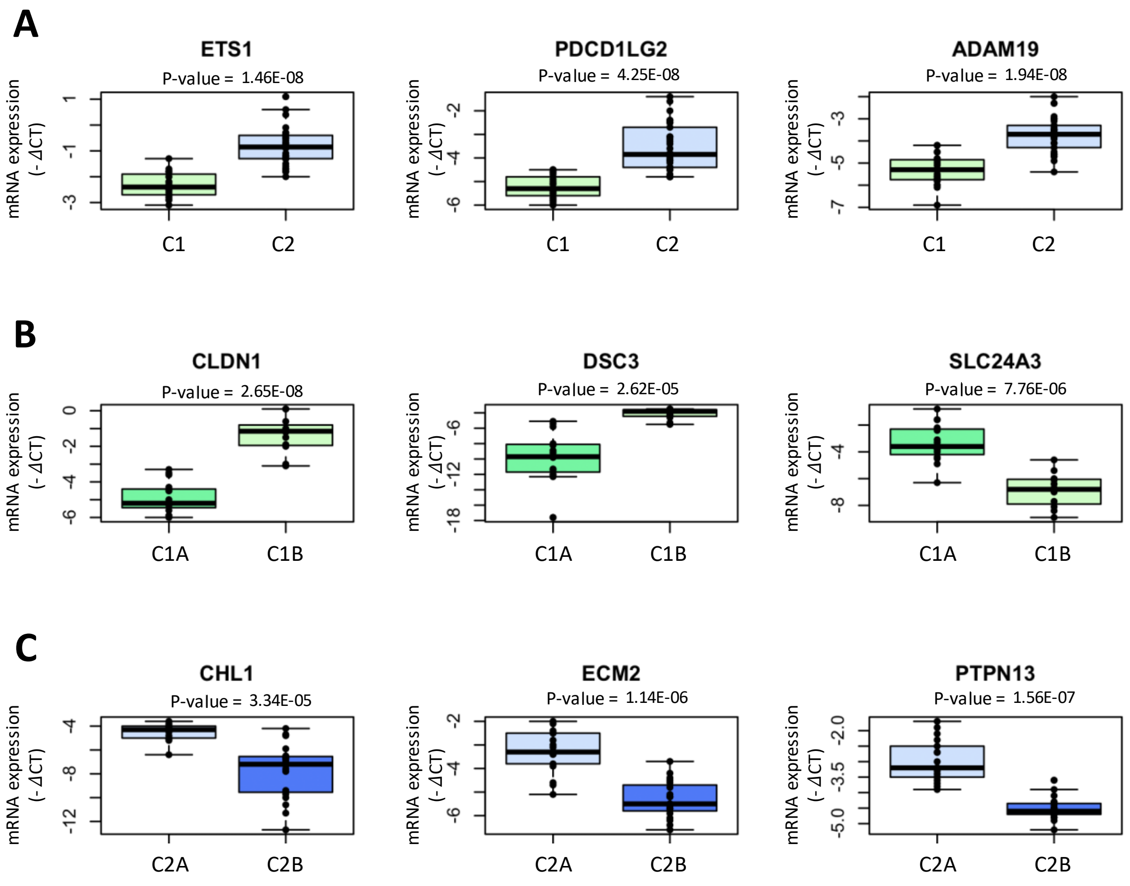

Figure S8 Gene expression of the 9-gene predictor.
(A-C) MRNA expression of the 9 genes of the predictor was measured by qRT-PCR. The boxplots indicate the distribution of mRNA levels in the Inserm series restricted to the 63 MPM previously annotated (Blum et al., 2019) for C1/C2 (A), C1A/C1B (B) and C2A/C2B (C) genes. For all boxplots, bottom and top of boxes are the first and third quartiles of the data, respectively, and whiskers represent the lowest (respectively highest) data point still within 1.5 interquartile range of the lower (respectively upper) quartile. Moderate t-tests were performed and FDR adjusted p-values are indicated above each dataset.

Reference

Blum, Y., Meiller, C., Quetel, L., Elarouci, N., Ayadi, M., Tashtanbaeva, D., Armenoult, L., Montagne, F., Tranchant, R., Renier, A., et al., 2019. Dissecting heterogeneity in malignant pleural mesothelioma through histo-molecular gradients for clinical applications. Nat Commun. 10, 1333.

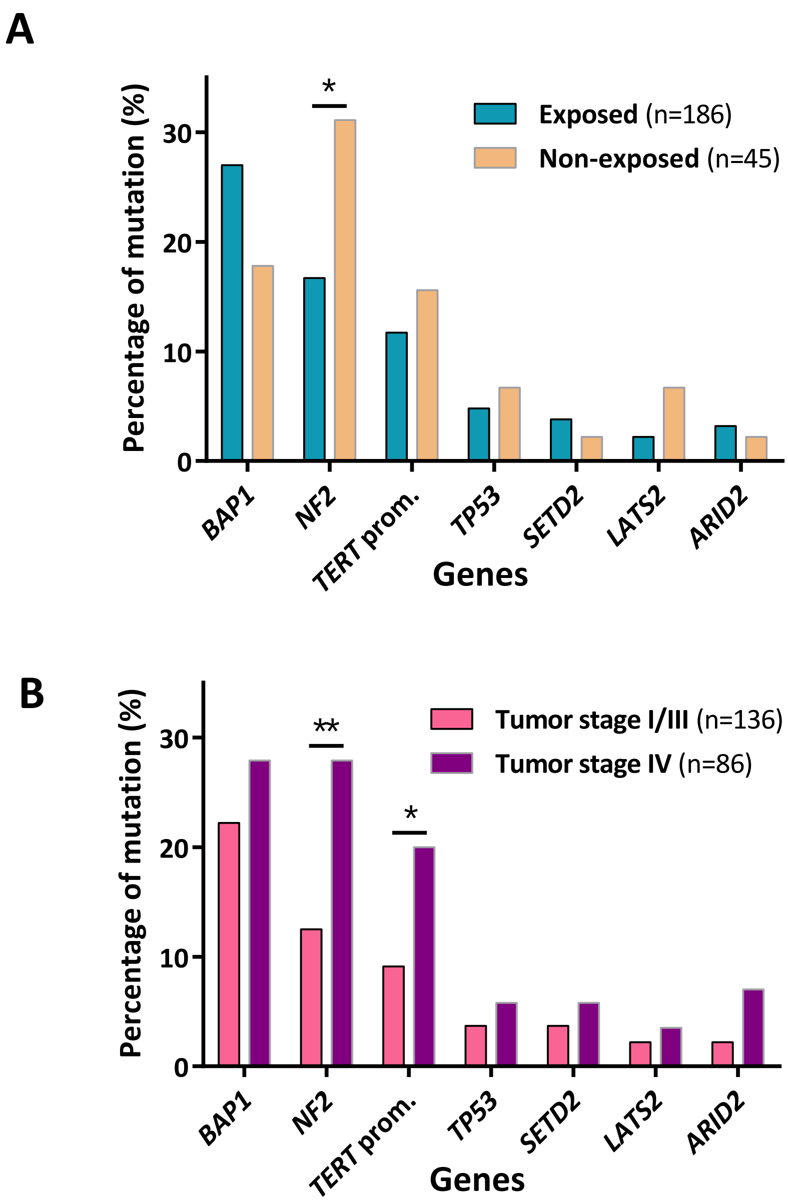

Figure S9 Associations between mutation profile and asbestos exposure status and tumor stage.

(A) Percentage of mutations in asbestos exposed and non-exposed patients. (B) Percentage of mutations in stage I/III and stage IV tumors. P-values were determined by the Fisher's exact tests (*: $p<0.05$; **: $p<0.01$; ***: $p<0.001$).

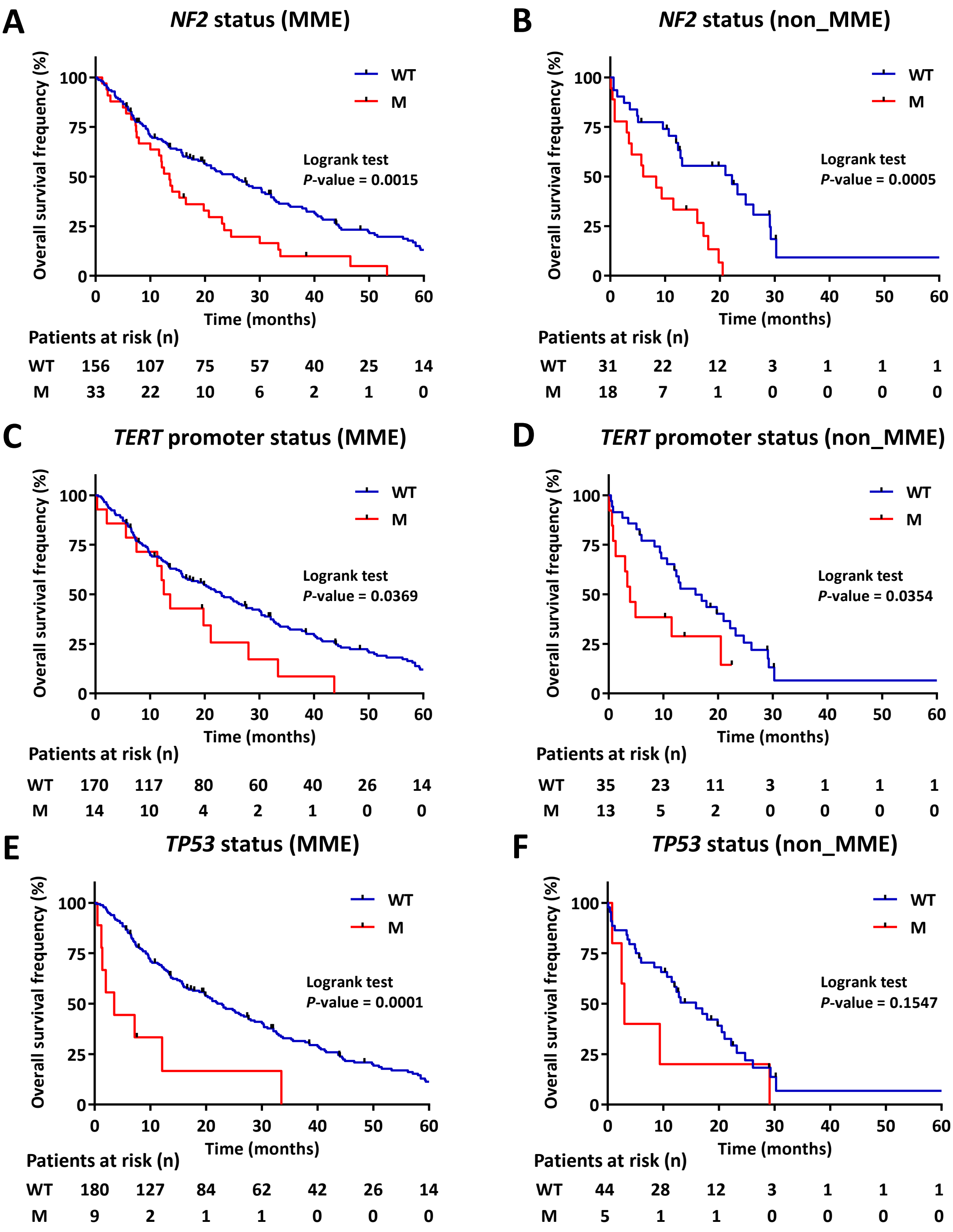

Figure S10 Associations between mutation profile and overall survival.

(A-F) Kaplan-Meier plots of overall survival in patients with wild-type (blue curve) or mutated (red curve) *NF2* gene (A and B), *TERT* promoter (C and D), *TP53* gene (E and F) MPM in MME (A, C and E) and non_MME samples (B, D and F). MMB, MMS and MMD were classified as non_MME. P-values were determined by the Log-rank test. MME: epithelioid MPM; MMB: biphasic MPM; MMS: sarcomatoid MPM; MMD: desmoplastic MPM.

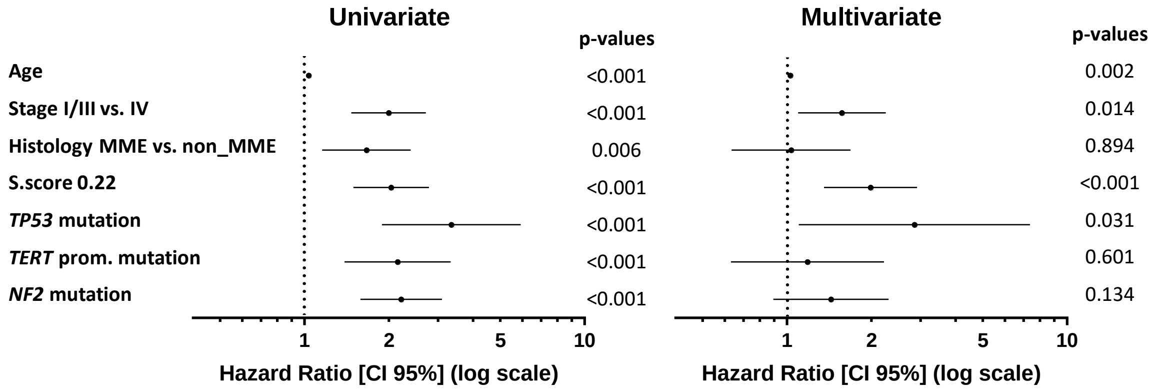

Figure S11 Prognostic value of *NF2*, *TERT* promoter and *TP53* mutations.
Univariate and multivariate Cox regression analyses of overall survival in MPM patients. Forest plots show hazard ratios (HR) and 95% confidence interval (CI) for overall survival according to age at diagnostic, tumor stage, histology, S.score based on a threshold of 0.22 and mutation status of *TP53*, *TERT* promoter and *NF2*. For histology, MMB, MMS and MMD were classified as non_MME. P-values of the Wald test for all variables are indicated at the right of each forest plot. MME: epithelioid MPM; MMB: biphasic MPM; MMS: sarcomatoid MPM; MMD: desmoplastic MPM; WT: wild type; M: mutated.

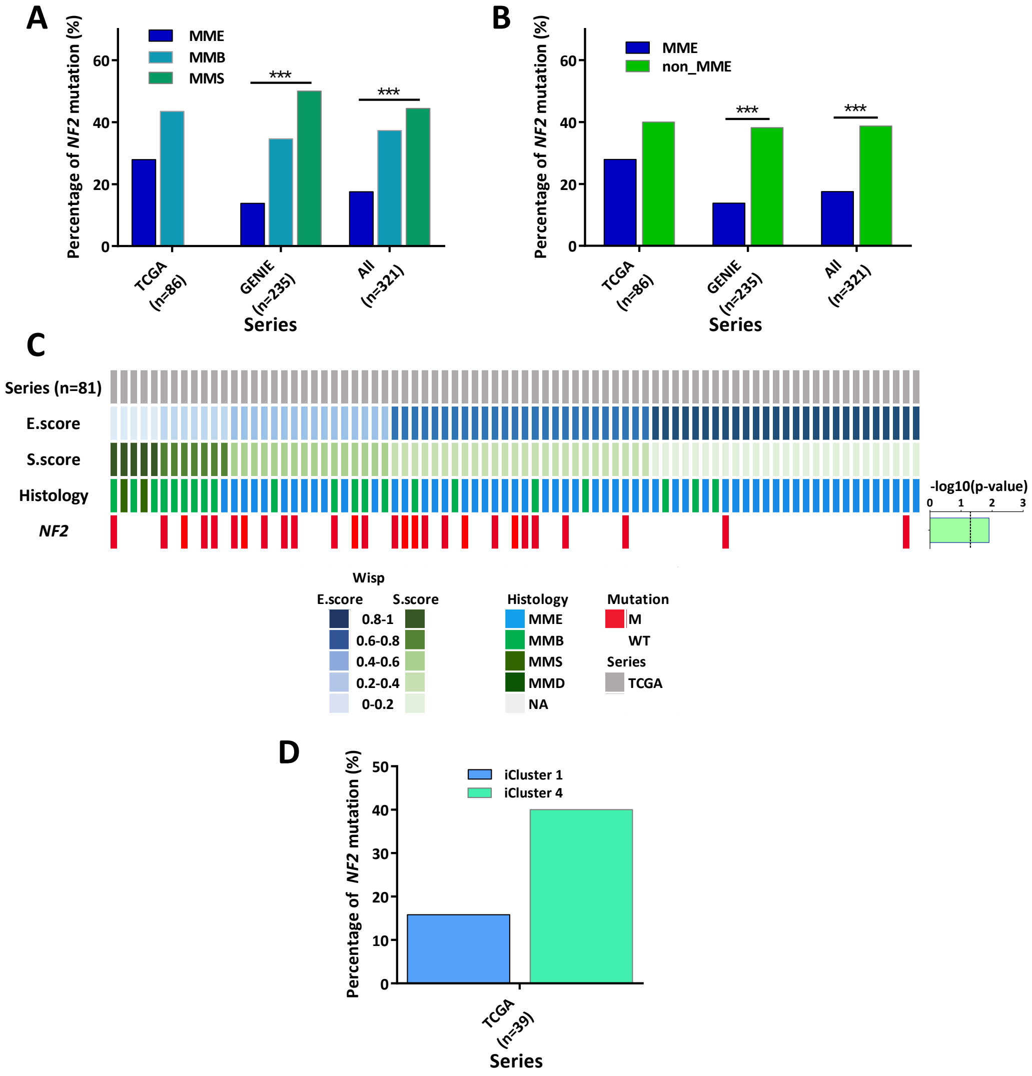

Figure S12 Associations between *NF2* mutation status including large deep deletions and, histologic and molecular subtypes or gradients.

NF2 large deep deletions status was only available for TCGA and GENIE series. (A and B) Associations between *NF2* mutation status and, MME, MMB and MMS (A) or MME and non_MME (B) histologic types in each MPM series and in all series. (C) Heat maps of the mutation status of *NF2* gene in tumor samples of TCGA series classified according to the E.score and S.score. Histogram on the right corresponds to -log10(p-value) of the Student's t test comparing for a specific gene the E.score or the S.score between MPM with or without any alterations. The black dashed line corresponds to a p-value threshold of 0.05. (D) Associations between *NF2* mutation status and molecular subtypes in TCGA series. P-values were determined by the Fisher's exact test (A, B and D) (*: p<0.05; **: p<0.01; ***: p<0.001). MME: epithelioid MPM; MMB: biphasic MPM; MMS: sarcomatoid MPM; MMD: desmoplastic MPM; WT: wild type; M: mutated.
